# *Tenebrio molitor* and *Hermetia illucens* Larvae Meals in Juvenile Nile Tilapia Diets: Performance, Digestibility and Hematological Responses Under *Streptococcus agalactiae* Challenge

**DOI:** 10.3390/insects17080762

**Published:** 2026-07-25

**Authors:** Maria Lucia Cocato, Eduardo Gianini Abimorad, Leandro Lopes Borges, Daniela Castellani, Pietro Ragozzino-Paulino, Jorge Eduardo de Souza Sarkis

**Affiliations:** 1 Lasers and Applications Center, Nuclear and Energy Research Institute (IPEN/CNEN—USP), Av. Prof. Lineu Prestes, 2242, São Paulo 05508-000, SP, Brazil; pietroragozzino01@gmail.com (P.R.-P.); jesarkis@ipen.br (J.E.d.S.S.); 2 Advanced Center for Research and Development of Continental Fish, Fisheries Institute, P.O. Box 1052, São José do Rio Preto 15025-970, SP, Brazil; abimorad@sp.gov.br (E.G.A.); leandrolb.biomar@gmail.com (L.L.B.); daniela.castellani@sp.gov.br (D.C.)

**Keywords:** Nile tilapia, *Tenebrio molitor*, *Hermetia illucens*, apparent digestibility, hematological profile, *Streptococcus agalactiae*

## Abstract

Insect meals are promising alternatives to conventional protein sources in aquafeeds, but comparative evidence in Nile tilapia (*Oreochromis niloticus*) remains limited. This study evaluated the effects of partially replacing an insect-free control diet with larvae meals from *Tenebrio molitor* and *Hermetia illucens* on growth performance, nutrient digestibility, hematological profile, and resistance to *Streptococcus agalactiae* in juvenile Nile tilapia. Fish were fed isonitrogenous and isoenergetic diets for 82 days: a control diet without insect meal and diets containing 10% dry matter of *T. molitor*, *H. illucens*, or a 1:1 mixture. Growth performance and somatic indices were not affected by dietary treatment. Lipid digestibility remained high and similar among diets; whereas, protein digestibility differed among diets and insect ingredients. Erythrocyte and total leukocyte counts were unchanged, but neutrophil and lymphocyte proportions differed among treatments. After intraperitoneal challenge with *S. agalactiae*, cumulative mortality was numerically lower in fish fed *T. molitor* or *H. illucens* than in the control group, although survival curves did not differ significantly. These findings indicate that both insect meals can be included at 10% in juvenile Nile tilapia diets without impairing growth, while influencing protein digestibility and leukocyte distribution.

## Simple Summary

Fish farming depends on feeds rich in protein, but traditional ingredients can be expensive and raise environmental concerns. Insects are being studied as a more sustainable alternative because they can provide high-quality nutrients while using fewer natural resources. In this study, we tested whether meals made from two insect species, the yellow mealworm and the black soldier fly, could be included in diets for juvenile Nile tilapia. Fish were fed these diets for 82 days, and we evaluated growth, nutrient use, blood health indicators, and survival after exposure to a bacterial infection that commonly affects tilapia farming. The insect-based diets did not reduce fish growth or overall body condition. Fat digestion remained high, while protein digestion varied depending on the insect source. Some blood cell proportions also changed, suggesting that the diets influenced aspects of the fish immune system. After bacterial challenge, fish fed the insect diets showed lower mortality values than the control group, although the differences were not statistically significant. Overall, the results support the use of these insect meals as promising sustainable ingredients for tilapia feeds.

## 1. Introduction

Nile tilapia (*Oreochromis niloticus*) is one of the most widely farmed freshwater fish species worldwide and a cornerstone of affordable animal protein, particularly in low- and middle-income countries. Recent reports from the Food and Agriculture Organization indicate that global aquaculture continues to expand and tilapia ranks among the main contributors to freshwater finfish production, with increasing relevance in emerging producer countries such as Brazil [1,2]. This rapid growth intensifies the demand for nutritionally adequate, cost-effective and environmentally sustainable feeds, while conventional protein sources such as fishmeal and soybean meal are constrained by price volatility, land and water use, and concerns about environmental footprints [3,4]. In this context, insect meals have attracted increasing attention as alternative feed ingredients for aquaculture. Several recent reviews highlight that insect larvae can convert low-value organic substrates into high-quality biomass rich in protein, lipids and micronutrients, with a more favorable environmental profile than many conventional ingredients [4,5,6]. Among the species considered, yellow mealworm (*Tenebrio molitor*) and black soldier fly (*Hermetia illucens*) have emerged as leading candidates due to their scalable production, balanced amino acid profile and promising performance in several farmed fish [4,7].

Recent experimental and review studies suggest that *T. molitor* meal can be used in fish diets without compromising growth, nutrient utilization or filet quality when included at appropriate levels [7,8]. In Nile tilapia, dietary inclusion of *T. molitor* larvae meal has been associated with improved filet quality traits and modulation of muscle metabolomic profiles, indicating potential benefits beyond simple protein replacement [9]. In addition, data from other species show that *T. molitor* meal can sustain growth and feed efficiency while affecting tissue lipid composition and antioxidant status, supporting its consideration as a versatile ingredient for aquafeeds [7,8]. The nutritional potential of *H. illucens* larvae for fish feeding has been even more extensively investigated. Meta-analyses and feeding trials indicate that black soldier fly meal can partially or totally replace fishmeal in diets for several freshwater and marine fish without impairing growth performance when formulations are properly balanced [3,4,7]. In Nile tilapia, replacement of fishmeal by *H. illucens* larvae meal has been shown to maintain or improve growth, feed utilization and body condition, while also influencing hematological parameters and mucosal responses [10,11,12]. More recently, studies using defatted *H. illucens* meal reported improved gut morphology, modulation of intestinal microbiota and enhanced resistance to pathogenic challenge in Nile tilapia, underscoring the relevance of insect meals for fish health as well as productivity [13,14].

Bacterial diseases remain a major bottleneck for sustainable tilapia aquaculture, and streptococcosis caused by *Streptococcus agalactiae* is among the most impactful conditions worldwide [15,16]. Outbreaks of *S. agalactiae* are associated with high cumulative mortalities, substantial economic losses and increasing concern about antimicrobial resistance due to the frequent use of antibiotics for disease control [15,17]. While vaccination and biosecurity measures are central to prevention, there is growing interest in nutritional approaches that support innate and adaptive defenses and improve survival under experimental challenge. In this regard, insect meals have been shown to influence immune-related biomarkers and survival in fish exposed to bacterial pathogens, including tilapia challenged with *Streptococcus iniae* [13,14,18].

Although the literature on insect meals in tilapia diets has expanded rapidly, most studies focus on a single insect species, use relatively short feeding periods, or assess only growth-related endpoints. Comparative information on diets containing *T. molitor* and *H. illucens* meals under a common experimental design, particularly including digestibility measurements, hematological profiling and survival after *S. agalactiae* challenge, is still limited [4,5,8]. Addressing this gap is important to clarify how different insect meals affect nutrient utilization, health status and disease outcomes in Nile tilapia, thereby guiding their rational inclusion in commercial feeds. Therefore, this study evaluated the effects of diets containing meals derived from *T. molitor* and *H. illucens* on the growth performance, nutrient digestibility, hematological profile and survival of Nile tilapia juveniles subjected to experimental *S. agalactiae* challenge. The findings aim to contribute to the evidence base for the safe and effective use of insect-based ingredients in tilapia aquaculture.

## 2. Materials and Methods

### 2.1. Experimental Facilities and Fish

**Figure 1:**
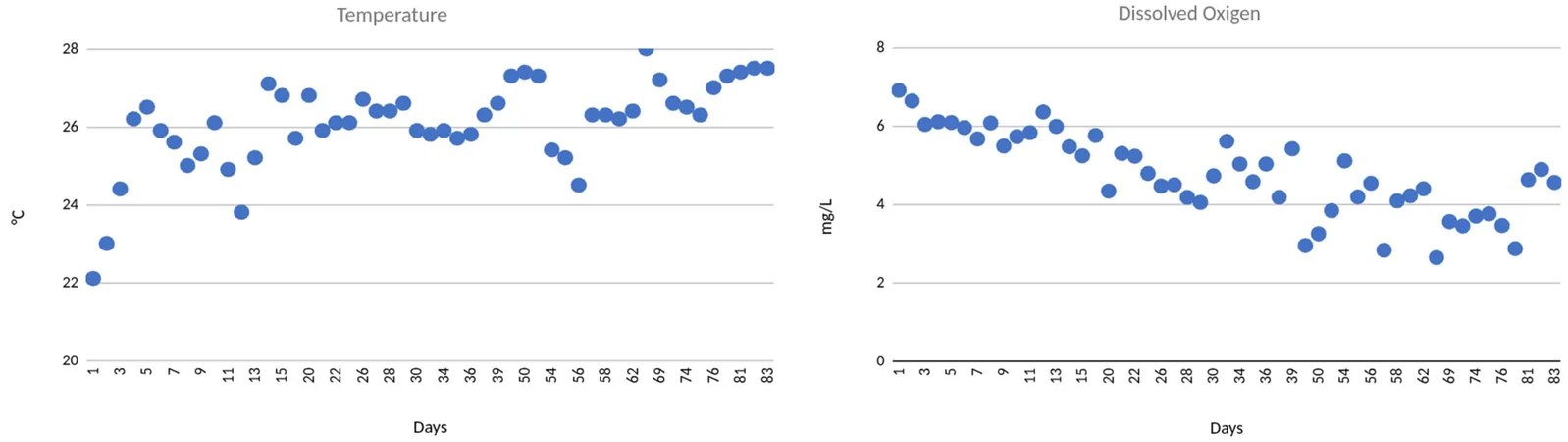
Water temperature and dissolved oxygen variation during the tilapia growth trial.

The feeding trial with Nile tilapia (*Oreochromis niloticus*) was carried out at the Laboratory of Nutrition and Aquaculture of the Fishing Institute (São José do Rio Preto, São Paulo, Brazil). The experimental system comprised 12 cylindrical tanks (Altamar, Jacareí, SP, Brazil, 310 L each), supplied individually by a recirculating water system equipped with a mechanical filter free of phyto- and zooplankton, a biological filter, thermostatic temperature control (26–28 °C) and continuous aeration, maintaining dissolved oxygen at ≥5 mg L^−1^. Throughout the experimental period, water temperature and dissolved oxygen were monitored daily (Figure 1); whereas, pH (6.35 ± 0.19), total ammonia (0.79 ± 0.96 mg L^−1^) and nitrite (0.69 ± 0.40 mg L^−1^) were monitored weekly. Filters of the recirculating system were cleaned weekly, and a partial water exchange (10%) was performed to maintain water quality.

Juvenile tilapia (20 g) were acquired from a commercial fish farm; fish were sex-reversed (♂) and not vaccinated, and were acclimated for approximately 20 days before selection for the feeding trial. Each experimental unit was stocked with 20 juvenile tilapia (initial body weight 28.79 ± 2.14 g at stocking). Before stocking, fish were anesthetized (eugenol, Biodinâmica Química e Farmacêutica, Ibiporã, PR, Brazil, 60 mg L^−1^), previously classified to obtain homogeneous groups, counted, individually weighed and measured, and then randomly allocated to the tanks. The feeding trial with insect-based diets lasted 82 days. An overview of the experimental design and sampling schedule is provided in Supplementary Figure S1.

All procedures involving fish were approved by the Animal Use Ethics Committee of the Instituto de Pesca (CEUA-IP, São José do Rio Preto, SP, Brazil) and complied with CONCEA guidelines (approval no. CEUA-IP 002/2022).

### 2.2. Experimental Diets

#### 2.2.1. Diet Formulation and Processing

Four experimental diets were formulated to be isonitrogenous (31.5% crude protein) and isoenergetic (13,288–13,572 kJ kg^−1^ digestible energy) to meet the nutritional requirements of the species [19]. The diets differed in the inclusion of insect larvae meals, which were incorporated at 10% (100 g kg^−1^ diet, dry matter basis) in the insect-based treatments: a control diet without insect ingredients (CON), a diet containing *Tenebrio molitor* larvae meal (TM), a diet containing *Hermetia illucens* larvae meal (HI), and a diet containing a 50:50 blend of *T. molitor* and *H. illucens* larvae meals (TMHI) (Table 1). The diets were formulated using conventional plant and rendered animal ingredients (e.g., soybean meal, poultry by-product meal, meat and bone meal, and blood meal) and common cereal energy sources (e.g., corn, broken rice, wheat bran), with ingredient proportions adjusted across treatments to maintain the target nutrient levels (Table 1).

To approximate field-like conditions, ingredients were sourced from commercial feed manufacturing supply chains; diets were ground through a 0.7 mm screen (hammer mill, Moinhos Vieria, Tatuí, SP, Brazil) and extruded as 3 mm pellets in a commercial extruder (Ferraz^®^ 62-e; Ferraz Máquinas, Ribeirão Preto, SP, Brazil; ~80 kg h^−1^).

#### 2.2.2. Chemical Characterization of Insect Meals

The chemical characterization of insect meals was performed at the Food Technology Institute (ITAL, Campinas—SP, Brazil), following standardized protocols under ABNT NBR ISO 9001 certification [20]. Crude protein was determined from total nitrogen according to AOAC Official Method 2001.11 [21], using a nitrogen-to-protein conversion factor of 6.25 (*N* × 6.25) to report crude protein for comparability with conventional feed-ingredient datasets. Total lipids were quantified by solvent extraction following the AOAC Official Method 920.39 [22], and results were expressed on a dry-matter basis. For fatty acid profiling, extracted lipids were converted to fatty acid methyl esters (FAME) following the AOAC Official Method 969.33 [23]. Amino acid composition was determined after hydrolysis and chromatographic quantification following the AOAC Official Method 982.30 for amino acid profiling [24]. Sulfur amino acids (cystine and methionine) were determined after oxidation/acid hydrolysis according to the AOAC Official Method 994.12 [25], and tryptophan was determined after alkaline hydrolysis according to the AOAC Official Method 988.15 [26]. Amino acids were reported as g 100 g^−1^ protein, as appropriate for diet formulation and ingredient evaluation. The proximate composition of the insect meals is shown in Table 2, fatty acid classes are presented in Table 3, and amino acid composition is reported in Table 4.

#### 2.2.3. Growth Performance and Somatic Indices

At the beginning and end of the feeding period, fish from each tank were anesthetized (eugenol, 60 mg L^−1^), individually weighed and measured. The following growth performance indicators were calculated at the tank level using standard equations for fish growth studies: feed intake, weight gain (WG), specific growth rate (SGR), feed conversion ratio (FCR), protein efficiency ratio (PER) and survival. Fish were fed four times daily to apparent satiation (ad libitum), avoiding feed leftovers in the water. Feed intake per tank was quantified by weighing feed containers when full and when leftovers were observed after a few days of feeding, followed by refilling with the experimental diets and reweighing. For the determination of somatic indices, three fish per replicate were euthanized at the end of the growth trial. Euthanasia was performed by eugenol overdose (200 mg L^−1^). Fish were dissected and viscera, liver and visceral fat were weighed for calculation of:

(1) $$Viscerosomatic\;index\;\left(VSI,\%\right)=\frac{viscera\;weight}{body\;weight}\;\times \;100$$

(2) $$Hepatosomatic\;index\;\left(HSI,\%\right)=\;\frac{liver\;weight}{body\;weight}\times 100$$

(3) $$Liposomatic\;index\;\left(LSI,\%\right)=\frac{visceral\;fat\;weight}{body\;weight}\times 100$$

#### 2.2.4. Apparent Digestibility

A digestibility trial was conducted in parallel with the growth trial using a separate experimental system. While insect meals were included at 10% of diet dry matter in the growth trial, ingredient digestibility was estimated using the substitution method, in which test diets contained 30% of each insect ingredient and 70% of a reference diet on a dry-matter basis [27]. A total of 360 juvenile Nile tilapia (mean body weight: 47.81 ± 7.13 g) were anesthetized with eugenol (60 mg L^−1^), sorted to obtain a homogeneous group, weighed, counted, and randomly distributed into twelve 300 L tanks, with 30 fish per tank. The experimental design consisted of four dietary treatments with three replicate tanks per treatment. Water temperature (27.9 ± 1.3 °C) and dissolved oxygen (4.86 ± 0.64 mg L^−1^) were monitored daily using a YSI 55 probe (Yellow Spring, OH, USA).

The reference diet used the same formulation as the control diet, and the test diets consisted of 70% reference diet and 30% test ingredient, namely *T. molitor* meal, *H. illucens* meal, or the *T. molitor* + *H. illucens* mixture. All digestibility diets contained 0.5% chromium(III) oxide (Cr_2_O_3_) as an inert marker for the indirect digestibility method, replacing corn in the formulation.

Following a 7-day adaptation and feeding period with the experimental diets, feces collection was initiated. Fish from each of the twelve feeding tanks were transferred to twelve feces-collection aquaria equipped with a modified Guelph system [28], consisting of cylindrical 120 L tanks with a conical bottom and a lower ball valve connected to Falcon-type tubes for recovery of sedimented feces. Feces were collected hourly from 07:00 am to 5:00 pm to minimize nutrient leaching and contamination.

At the end of each daily collection period, fish were returned to their respective feeding tanks and maintained on the experimental diets for an additional 7 days before a new feces-collection cycle was conducted. This feeding–collection procedure was repeated three times. Fecal samples obtained from each experimental unit across the three collection cycles were pooled to provide sufficient material for chemical analyses. The pooled samples were subsequently dried in a forced-air oven at 55 °C for 48 h, ground using a mortar and pestle, manually separated from scales and debris, and stored until laboratory analyses.

After determining Cr_2_O_3_, crude protein and ether extract in the reference diet, test diets and feces, apparent digestibility coefficients (ADC) of the diets were calculated according to Nose [29]:

(4) $$ADC\;\left(\%\right)=\;100\;\times \;\left[1\;-\;\left(\frac{\%Cr_{2}O_{3}diet}{\%Cr_{2}O_{3}\;feces}\right)\times \left(\frac{\%N\;diet}{\%N\;feces}\right)\right]\;$$

where $Cr_{2}O_{3}diet$ and $Cr_{2}O_{3}\;feces$ are chromium oxide concentrations in diet and feces, respectively, and N diet and N feces are the concentrations of the nutrient of interest (crude protein or ether extract) in diet and feces.

Digestibility of the test insect ingredients was calculated using the equation described by Bureau et al. [27]:

(5) $${ADC}_{I}\left(\%\right)=\;{ADC}_{T}+\left(0.7\;D_{R}/0.3\;D_{I}\right)\;\left({ADC}_{T}-{ADC}_{R}\right)\;$$

where ADC_I_ is the ADC of the test ingredient; ADC_T_ is the ADC of the test diet; ADC_R_ is the ADC of the reference diet; D_R_ is the % nutrient of the reference diet mash; D_I_ is the % nutrient of the test ingredient; 0.3 is the proportion of the test ingredient in the test diet mash; and 0.7 is the proportion of the reference diet mash in the test diet mash.

#### 2.2.5. Hematological Analyses

Hematological sampling was performed immediately after the growth trial. Two fish were randomly selected from each tank (six fish per diet). Blood was collected by caudal vessel puncture using 3 mL disposable syringes, and aliquots were transferred to 500 µL microtubes containing EDTA as anticoagulant (BD Microtainer^®^, Becton, Dickinson and Company, Franklin Lakes, NJ, USA). Erythrocyte counts, hematocrit, total leukocyte and thrombocyte estimates, and differential leukocyte counts (neutrophils, lymphocytes, monocytes, eosinophils and basophils) were determined. Red blood cells were counted in a Neubauer chamber (Marienfeld Superior, Lauda-Königshofen, Germany), and hematocrit was determined using the microhematocrit method described by Goldenfarb et al. [30].

Blood smears were prepared, air-dried, stained, and examined under light microscopy for differential leukocyte counts, with at least 200 leukocytes counted per slide. Total leukocyte counts were estimated using an indirect smear-based method by counting approximately 2000 erythrocytes per smear and deriving leukocyte numbers from the leukocyte:erythrocyte ratio. Differential leukocyte counts were performed on smears stained with Rosenfeld stain.

#### 2.2.6. *Streptococcus agalactiae* Challenge

Before the main challenge test, a preliminary trial was conducted to determine the median lethal dose (LD_50_) of a pathogenic *Streptococcus agalactiae* strain in Nile tilapia through intraperitoneal injection of 100 µL bacterial suspensions at graded concentrations. For the bacterial preparation, the isolate was plated on Brain Heart Infusion (BHI) agar (HiMedia Laboratories Pvt. Ltd., Mumbai, India) supplemented with 5% blood (30 °C, 24 h); colonies were suspended in sterile 0.85% saline to an optical density of 0.330 at 600 nm (stock suspension), followed by 10-fold serial dilutions and CFU confirmation by plate counts.

Fish were anesthetized in buckets with eugenol (15 mg L^−1^) before injection; 100 µL of inoculum was injected intraperitoneally using insulin syringes, and fish were returned to tanks until full recovery of normal activity. Mortality was recorded daily until three consecutive days without deaths were observed, and LD_50_ was defined as the dose producing ~50% cumulative mortality. The LD_50_ was determined as 1.65 × 10^5^ CFU.

In the main challenge assay, fish previously fed the experimental diets were injected intraperitoneally with 100 µL of the *S. agalactiae* suspension at the LD_50_ dose determined in the preliminary test (1.65 × 10^5^ CFU). Two independent challenge blocks were conducted under the same protocol on different dates (Infection 1 and Infection 2). A negative control group (CN; derived from the CON treatment) received sterile 0.85% saline injection, and an infected positive control group (CP) was included to verify challenge validity. Mortality was monitored daily throughout the observation period, and cumulative mortality (%) was calculated as (cumulative deaths/total fish) × 100. The infection course typically ended around day 10 post-inoculation, defined as three consecutive days without mortality. Potential block effects were assessed by comparing final mortality proportions between Infection 1 and Infection 2; as no evidence of block differences was detected, data were pooled for presentation and analysis (Table S1).

#### 2.2.7. Statistical Analysis

Data on growth performance, somatic indices and apparent digestibility were analyzed by one-way analysis of variance (ANOVA) with dietary treatment as the fixed factor, using the tank as the experimental unit. When significant differences were detected (*p* < 0.05), means were compared using Tukey’s multiple comparison test. Hematological variables were analyzed using a mixed model in which dietary treatment was included as a fixed effect and tank (nested within diet) as a random effect, with individual fish treated as subsamples within tank. When significant effects were detected (*p* < 0.05), means were compared using Tukey’s test. Statistical analyses were performed using SAS software, version 9.0 (SAS Institute Inc., Cary, NC, USA).

Post-challenge survival was analyzed using Kaplan–Meier time-to-event curves based on daily mortality records, and differences among dietary treatments were tested using the log-rank test (*p* < 0.05). Potential challenge-block effects (Infection 1 vs. Infection 2) were assessed by comparing final mortality proportions between blocks; as no evidence of block differences was detected, data were pooled for presentation and analysis (Table S1). Cumulative mortality was additionally summarized descriptively as the proportion of dead fish per treatment over the 13-day observation period.

## 3. Results

### 3.1. Growth Performance

Growth performance data for Nile tilapia are summarized in Table 5. No statistically significant differences among diets were detected for initial body weight (*p* = 0.2802), final body weight (*p* = 0.7388), weight gain (*p* = 0.7464), specific growth rate (SGR; *p* = 0.7110) or feed intake (*p* = 0.3043). Initial body weight ranged from 28.28 ± 0.28 g (TM) to 29.22 ± 0.32 g (TMHI); whereas, final body weight ranged from 248.31 ± 13.31 g (CON) to 260.36 ± 6.35 g (TMHI) (Table 5).

Feed conversion ratio did not differ significantly among dietary treatments (*p* = 0.0525), indicating that the inclusion of insect meals did not impair feed efficiency under the present experimental conditions (Table 5). Overall, partial replacement of the control diet by *Tenebrio molitor* and/or *Hermetia illucens* larvae meals at 10% diet did not impair growth performance of Nile tilapia juveniles under the present experimental conditions.

### 3.2. Survival and Somatic Indices

Survival and somatic indices are presented in Table 6. Survival was high across treatments (93.3 ± 5.8% to 100.0 ± 0.0%) and did not differ among diets (*p* = 0.7797). Condition factor (*p* = 0.3832), viscerosomatic index (VSI; *p* = 0.8487) and hepatosomatic index (HSI; *p* = 0.5478) were also not affected by dietary treatment (*p* > 0.05), with comparable mean values among CON, TM, HI and TMHI (Table 6). Liposomatic index (LSI) also did not differ among diets (*p* = 0.0544), with mean values ranging from 1.72 ± 0.49% in HI to 2.40 ± 0.06% in TMHI (Table 6).

### 3.3. Apparent Digestibility Coefficients

Apparent digestibility coefficients for crude protein (ADC-CP) and ether extract (ADC-EE) of the experimental diets and insect ingredients are shown in Table 7. Ingredient digestibility values were estimated using the substitution method (test diets containing 30% insect ingredient and 70% reference diet). Dietary treatment significantly affected diet ADC-CP (*p* = 0.0134), with fish fed TM showing a lower ADC-CP (83.60 ± 1.87%) than the other treatments; whereas, CON (86.95 ± 0.71%), HI (87.31 ± 0.87%) and TMHI (85.71 ± 0.42%) did not differ from each other (Table 7). In contrast, diet ADC-EE did not differ among treatments (*p* = 0.9132), ranging from 86.76 ± 1.66% (HI) to 88.95 ± 0.91% (TMHI) (Table 7).

At the ingredient level, ADC-CP differed among insect meals (*p* = 0.0265). *H. illucens* meal showed the highest value (88.16 ± 2.90%), *T. molitor* meal the lowest (75.79 ± 6.22%), and the mixed insect meal (TMHI) intermediate digestibility (82.83 ± 1.41%) (Table 7). Ingredient ADC-EE did not differ among insect meals (*p* = 0.8343), with values of 88.70 ± 4.73% (TM), 85.69 ± 5.52% (HI) and 93.48 ± 2.93% (TMHI) (Table 7). Overall, lipid digestibility was high across insect meals; whereas, protein digestibility ranged from 75.79 to 88.16% under the present experimental conditions.

### 3.4. Hematological Parameters

Red blood cell (erythrocytes) and total leukocyte counts did not differ among dietary treatments (*p* = 0.3332 and *p* = 0.3616, respectively; Table 8). However, differential leukocyte counts showed a dietary effect on the relative proportions of neutrophils (*p* = 0.0247) and lymphocytes (*p* = 0.0172) (Table 9). Fish fed the control diet exhibited a higher percentage of neutrophils (32.33 ± 5.75%) and a lower percentage of lymphocytes (55.33 ± 3.62%) compared with fish fed HI (22.50 ± 5.57% neutrophils; 66.33 ± 6.41% lymphocytes) and TMHI (21.83 ± 8.11% neutrophils; 65.00 ± 7.77% lymphocytes); whereas, TM showed intermediate values (Table 9). No significant differences were detected for monocytes (*p* = 0.5101), eosinophils (*p* = 0.8832) or basophils (*p* = 0.0628) (Table 9).

### 3.5. Streptococcus agalactiae Challenge and Mortality

Daily mortality data are provided in Supplementary Table S2; whereas, cumulative mortality, survival, confidence intervals and the observation period are summarized in Table 10. Mortality data are presented as pooled outcomes from two independent challenge blocks (Infection 1: *n* = 24 fish per diet; Infection 2: *n* = 22 fish per diet; total *n* = 46 fish per diet) conducted under the same protocol. Final mortality proportions were comparable between Infection 1 and Infection 2 (*p* > 0.05), supporting pooling of both challenge blocks (Table S1). The observation period lasted 13 days post-challenge (Table 10). Kaplan–Meier survival curves derived from daily mortality records did not detect differences among dietary treatments (log-rank test, *p* = 0.592), and thus between-group differences in cumulative mortality are interpreted descriptively under the present experimental conditions. The negative control (CN) showed 4/46 deaths (8.7%); whereas, the positive control (CP) showed 30/46 deaths (65.2%) over the observation period (Table S1). Cumulative mortality was highest in fish fed the control diet (CON: 65.2%, 30/46; 95% CI: 50.8–77.3), corresponding to a survival of 34.8% (Table 10). Among the insect-based diets, cumulative mortality was numerically lower in HI (50.0%, 23/46; 95% CI: 36.1–63.9) and TM (52.2%, 24/46; 95% CI: 38.1–65.9); whereas, TMHI showed an intermediate value (60.9%, 28/46; 95% CI: 46.5–73.6) (Table 10). The temporal pattern of deaths is provided in Table S2. Most mortality occurred during the early post-challenge phase, particularly between days 3 and 5, after which deaths became sporadic and cumulative mortality curves approached a plateau toward the end of the observation period (Table S2).

## 4. Discussion

### 4.1. Growth Performance and Somatic Responses

The present study showed that partial replacement of the control diet by *Tenebrio molitor* and/or *Hermetia illucens* larvae meals at 10% inclusion did not impair growth performance of Nile tilapia juveniles under the present experimental conditions. Final body weight, weight gain, specific growth rate (SGR), feed intake and feed conversion ratio (FCR) did not differ among diets (*p* > 0.05) (Table 5). Overall, fish showed strong productive responses across treatments, with SGR values of approximately 2.76–2.82% day^−1^, FCR values of 1.15–1.25 and survival above 93%. These values indicate efficient growth and feed utilization for juvenile Nile tilapia under controlled feeding conditions and support the nutritional adequacy of the experimental diets.

These findings are consistent with previous studies showing that *T. molitor* and *H. illucens* meals can be incorporated into tilapia feeds without compromising growth performance when inclusion levels are appropriate and diets are properly balanced [4,10,31,32,33,34]. In Nile tilapia, *H. illucens* larvae meal has been tested as a fishmeal replacer without detrimental effects on growth and feed utilization [10,31], while graded substitution designs further support its feasibility in nutritionally balanced diets [32]. Similarly, studies using *T. molitor* in tilapia diets, including biofloc systems and digestibility-focused trials, indicate that this ingredient can support adequate productive performance when formulation constraints are addressed [34,35]. Therefore, the absence of negative effects in the present study suggests that, at 10% inclusion, both insect meals acted as nutritionally compatible ingredients rather than as performance-enhancing additives, as also indicated in broader syntheses on insect meal use in aquafeeds [33,36,37,38].

Somatic indices further support the hypothesis that insect meal inclusion at 10% did not induce major alterations in body condition, organ allocation or visceral fat deposition. Condition factor, viscerosomatic index (VSI), hepatosomatic index (HSI) and liposomatic index (LSI) were not affected by dietary treatment (*p* > 0.05) (Table 6). This pattern is consistent with tilapia trials using *H. illucens* meal that reported stable body condition and somatic responses under practical formulations [10,33]. Although mean LSI values varied among treatments, this response remained below the significance threshold and should therefore be interpreted as descriptive. Overall, the growth and somatic responses observed here add to the evidence that *T. molitor* and *H. illucens* meals are technically feasible ingredients for juvenile Nile tilapia feeds at low-to-moderate inclusion levels, provided that diets are formulated to meet nutrient requirements and maintain adequate amino acid balance and digestible energy [4,10,32,34]. However, because only one inclusion level was evaluated, future studies using graded inclusion levels are warranted to establish optimal inclusion thresholds and to determine whether higher dietary incorporation may elicit more pronounced physiological, immunological or productive responses in Nile tilapia.

### 4.2. Nutrient Digestibility of Tenebrio molitor and Hermetia illucens Meals

The apparent digestibility coefficients (ADC) obtained in the present study indicate that both insect meals supported high nutrient utilization by Nile tilapia. Protein digestibility was more sensitive to diet and ingredient differences than lipid digestibility, a pattern commonly observed when comparing protein-rich ingredients that differ in non-protein fractions, processing history and nutrient accessibility [35,39]. It is important to note that insect meals were included at 10% in the growth trial; whereas, ingredient ADC values were estimated using the substitution method [27]. Therefore, diet-level ADC values are more directly related to the growth trial; whereas, ingredient-level ADC values provide comparative information on the digestibility of each insect meal.

At the diet level, ADC-CP differed among treatments, with the TM diet showing lower protein digestibility than CON, HI and TMHI; whereas, ADC-EE did not differ among diets and remained high across treatments (Table 7). This indicates that Nile tilapia maintained a consistent capacity to digest dietary lipids across formulations, as also reported in studies evaluating practical diets and insect-derived ingredients in tilapia [10,40,41,42]. The lower ADC-CP observed for the TM diet did not result in impaired growth performance or poorer FCR at 10% inclusion, suggesting that the overall digestible nutrient supply remained adequate when diets were properly formulated. Conversely, although the HI diet presented a higher mean FCR, this response was not accompanied by lower ADC-CP or ADC-EE values. Therefore, the FCR pattern observed in HI cannot be explained solely by the apparent digestibility of crude protein or ether extract. If confirmed in adequately powered studies, a higher FCR in fish fed HI could indicate lower overall feed efficiency, potentially related to factors not fully captured by the digestibility assay, such as amino acid balance, nutrient availability, ingredient matrix effects, energy partitioning, or other aspects of nutrient utilization. Importantly, FCR did not differ significantly among diets, and this interpretation should therefore be considered descriptive rather than evidence of a treatment effect.

At the ingredient level, ADC-CP also differed among insect meals. *H. illucens* meal showed the highest protein digestibility, *T. molitor* meal the lowest, and the mixed insect meal an intermediate value (Table 7). In contrast, ingredient ADC-EE did not differ among insect meals and remained high. These findings support the interpretation that protein digestibility is a major source of variation among insect ingredients, while lipid utilization may remain consistently high across insect sources when diets are balanced. Similar patterns have been reported in tilapia digestibility screening assays and feeding trials including *H. illucens* meal [10,34,35,42].

Variation in insect-meal protein digestibility may be related to ingredient composition and processing, including non-protein fractions such as chitin, degree of defatting and physicochemical properties that can influence nutrient accessibility and enzyme–substrate interactions [43,44]. In Nile tilapia, chitin can be digested to some extent, but its effects on nutrient digestibility may vary according to inclusion level, processing and dietary context [44]. In this sense, the lower ADC-CP observed for TM does not preclude its use in tilapia feeds, particularly because growth performance was maintained in the present study. However, it highlights the importance of ingredient characterization and formulation adjustments, including amino acid balancing and consideration of processing effects, when insect meals are used as feed ingredients [39]. Overall, the high ADC values observed, especially for lipid digestibility, are consistent with the capacity of Nile tilapia to utilize insect-derived nutrients and support the inclusion of *T. molitor* and *H. illucens* meals at 10% in balanced juvenile tilapia diets.

### 4.3. Hematological Responses and Immunomodulation

Hematological profiles are widely used as practical indicators of fish health and can reflect nutritional adequacy and general physiological status [45]. In the present study, erythrocyte counts and total leukocyte counts were not affected by dietary treatment (*p* > 0.05; Table 8), indicating that inclusion of *T. molitor* and *H. illucens* meals at 10% did not compromise basic hematological homeostasis in Nile tilapia juveniles. Comparable outcomes have been reported for Nile tilapia fed *T. molitor* meal under intensive rearing conditions, where baseline hematological parameters remained within physiological ranges at similar inclusion levels [34]. Similar responses have also been described in tilapia trials using *H. illucens* meal in balanced formulations [10].

Although total leukocyte counts did not differ among treatments, the differential leukocyte profile showed diet-related differences in the relative proportions of neutrophils and lymphocytes (Table 9). Fish fed the control diet had higher neutrophil percentages and lower lymphocyte percentages than fish fed HI and TMHI; whereas, TM showed an intermediate profile. No significant differences were observed for monocytes, eosinophils or basophils (*p* > 0.05). Because these changes occurred without differences in total leukocyte counts, they suggest a shift in leukocyte distribution rather than generalized leukocytosis or leukopenia.

This pattern may indicate a modest diet-associated modulation of leukocyte profiles under basal conditions, but it should not be interpreted as direct evidence of improved immune competence. Previous studies and reviews have reported that insect-derived ingredients can influence immune-related endpoints in fish, including hematological and mucosal responses, without necessarily altering core hematological variables [4,5,10,46]. Such effects have been partly attributed to non-protein fractions and bioactive components of insect meals, including chitin-related compounds and lipid fractions, although their responses depend on insect species, processing, inclusion level and diet formulation [4,43,46,47,48].

Therefore, the hematological results of the present study support the safety of 10% inclusion of *T. molitor* and *H. illucens* meals and suggest limited effects on leukocyte distribution. However, because functional immune assays, oxidative-stress biomarkers, cytokine or gene-expression analyses and microbiota assessments were not performed, the biological relevance of these leukocyte shifts remains uncertain. Future studies combining differential leukocyte profiles with functional and molecular endpoints would help clarify whether these changes translate into measurable immunological benefits or improved disease resistance [4,5,43,46].

### 4.4. Resistance to Streptococcus agalactiae

Streptococcosis caused by *Streptococcus agalactiae* is among the most relevant bacterial diseases affecting Nile tilapia production and is frequently associated with acute outbreaks, high mortality and substantial economic losses to producers [49,50]. Beyond pathogen factors, host outcomes are strongly modulated by farming conditions, such as temperature and stocking density, pathogen strain and virulence, challenge model and the baseline physiological and immune status of the fish, which helps explain why mortality can vary considerably among studies and production settings [49,51]. Consequently, nutritional strategies that support fish robustness and post-challenge resilience are of practical interest as complementary tools alongside vaccination, biosecurity and husbandry adjustments [50,52].

In the present study, pre-challenge feeding with insect-based diets did not significantly affect survival after *S. agalactiae* challenge, as indicated by Kaplan–Meier analysis and the log-rank test (*p* = 0.592). Although cumulative mortality was lower in TM and HI than in CON, these differences were not statistically significant and should therefore be interpreted descriptively. The difference in cumulative mortality between CON and the individual insect-meal diets was approximately 13–15 percentage points, with 65.2% mortality in CON compared with 52.2% in TM and 50.0% in HI; whereas, TMHI showed an intermediate value of 60.9%. These descriptive differences may warrant confirmation in adequately powered challenge trials, particularly because even modest reductions in cumulative mortality can translate into meaningful gains in harvestable biomass and lower unit production costs in intensive systems where disease events can rapidly erode margins [50,53,54]. Farm-level economic appraisals of streptococcosis control strategies illustrate how shifts in mortality can materially affect profitability at the production-cycle level [50,53].

The descriptive mortality pattern observed here is consistent with previous studies showing that dietary strategies can modulate immune responses and disease outcomes in Nile tilapia exposed to *Streptococcus* spp. Diets supplemented with functional ingredients, including yeast-derived compounds, plant-based additives, organic acids and other immunonutritional strategies, have been evaluated in tilapia challenged with *S. agalactiae*, with reported effects on inflammatory responses, hematological variables, intestinal morphology and/or survival depending on the additive and experimental model [53,54]. These studies support the general concept that nutrition can influence host condition during streptococcosis, but they also show that post-challenge survival is a multifactorial endpoint and may not respond consistently across dietary interventions.

Evidence specifically involving insect meals further supports their potential to modulate immune-related endpoints in fish. In Nile tilapia, Tippayadara et al. [10] reported that replacement of fish meal by *H. illucens* larvae meal was associated with stable growth, survival and core hematological variables, while improving skin mucus lysozyme and peroxidase activities. Similarly, Alves et al. [18] showed that Nile tilapia fed *Zophobas morio* meal under lipopolysaccharide challenge maintained growth performance and showed modulation of innate immune indicators, including lysozyme activity and the alternative complement system, before and after immune stimulation. Agbohessou et al. [55] also reported that fatty acid-enriched dipteran-based meals affected digestive and immunological responses in Nile tilapia juveniles, suggesting that insect-derived ingredients may influence immune physiology beyond their role as protein sources. Together, these studies indicate that insect meals can affect systemic and mucosal immune-related responses in tilapia, although the magnitude and direction of these effects depend on insect species, processing, inclusion level, formulation and immune stimulus.

More directly related to bacterial challenge, Abd El-Gawad et al. [14] reported that defatted *H. illucens* meal improved hemato-immunological, antioxidant and inflammatory-related responses in Nile tilapia challenged with *Streptococcus iniae*. Although *S. iniae* is not the same pathogen used in the present study, both agents are important causes of systemic streptococcosis in tilapia and involve overlapping host-response pathways [49]. Therefore, that study provides relevant support for the biological plausibility that *H. illucens* meal may influence host condition under streptococcal infection pressure, while still requiring caution when extrapolating to *S. agalactiae*.

Evidence across other fish models also indicates that *T. molitor* meal can modulate innate immune effectors and antibacterial activities that may be relevant to bacterial-disease outcomes. Feeding European sea bass diets containing *T. molitor* larvae meal increased lysozyme antibacterial activity and altered humoral inflammatory-related markers, including myeloperoxidase and nitric oxide, consistent with mild immunostimulation rather than immunosuppression [56]. Likewise, in juvenile yellow catfish, graded inclusion of *T. molitor* meal enhanced immune and antioxidant indicators and was evaluated in the context of disease-resistance responses, supporting the concept that mealworm-based formulations can influence host defense readiness under infection pressure [57]. In mandarin fish, dietary *T. molitor* inclusion has also been associated with changes in lysozyme activity and antioxidant enzyme activity, reinforcing that mealworm meal may affect innate immune and oxidative-status markers in fish [58]. In red seabream, defatted T. molitor larvae meal also improved growth performance and disease resistance after bacterial challenge, further supporting the potential functional relevance of mealworm-based diets in fish [59]. These studies provide biological support for the descriptive mortality pattern observed for TM in the present work, but they do not demonstrate a causal protective effect against *S. agalactiae*.

The leukocyte shifts observed before challenge may also support a cautious interpretation of diet-associated immune modulation. Fish fed HI and TMHI showed higher lymphocyte proportions and lower neutrophil proportions than CON; whereas, TM showed an intermediate profile. In the context of the descriptive mortality pattern, these changes are compatible with the broader idea that insect meals can influence basal immune status [4,46]. However, because total leukocyte counts were not affected and the present study did not measure lysozyme or complement activity, oxidative-stress biomarkers, cytokine expression, microbiota composition, pathogen load or tissue-level pathology, mechanistic links among diet composition, leukocyte distribution and survival outcomes remain hypothetical.

Several insect-derived components have been proposed as contributors to immune-related responses in fish. Chitin and chitin-derived fractions may interact with innate and mucosal immune pathways, while *H. illucens*-derived lipid fractions, including medium-chain fatty acids such as lauric acid, have been discussed in relation to antimicrobial activity and host–microbe interactions [4,43,46,47,60,61,62]. In vitro studies have also reported antimicrobial activity of lipids extracted from *H. illucens* and *T. molitor*, supporting the plausibility that insect lipid fractions may contribute to microbial modulation under some conditions [61,62]. Nevertheless, the present study did not evaluate chitin fractions, lipid bioactivity, gut microbiota or pathogen load. Therefore, these mechanisms should be regarded as plausible explanations based on previous literature, but not as mechanisms demonstrated in the present experiment.

The intermediate cumulative mortality observed in TMHI should not be interpreted as evidence of antagonism or reduced efficacy of the blend, because survival curves did not differ statistically and the experiment was not designed or powered to resolve small differences among insect-based diets. A mixed-insect formulation may generate responses that differ from single-source meals because the relative contribution of functional fractions, nutrient profile and non-protein components can change when ingredients are blended. However, without direct measurements of immune function, gut microbiota, pathogen burden or intestinal responses, the intermediate TMHI outcome should be considered descriptive rather than mechanistically explained.

Accordingly, the present evidence supports a neutral effect of 10% insect meal inclusion on *S. agalactiae* resistance, with descriptive mortality patterns that may justify further investigation. Future work should combine challenge trials with integrated immune and microbiological endpoints, including lysozyme and complement activity, mucosal markers, gut histology, microbiota profiling, pathogen-load quantification and tissue pathology. Given the economic relevance of streptococcosis, future studies should also consider sample sizes powered to detect smaller but commercially meaningful differences in survival [50,53].

## 5. Conclusions

This study demonstrates that replacing an insect-free control diet with *Tenebrio molitor* and/or *Hermetia illucens* larvae meals at 10% inclusion on a dry-matter basis is nutritionally feasible for juvenile Nile tilapia. At this inclusion level, growth performance and major somatic indices were maintained, and feed conversion ratio and liposomatic index did not differ among diets (*p* > 0.05). Digestibility results further supported the technical suitability of both ingredients, showing consistently high lipid digestibility, while protein digestibility differed among diets and insect ingredients. Nevertheless, the overall digestible nutrient supply remained sufficient to sustain growth under the present experimental conditions. Hematological profiles were largely stable, and post-challenge outcomes should be interpreted descriptively, as no significant differences in survival were detected after *Streptococcus agalactiae* challenge.

The shifts observed in leukocyte distribution in insect-fed fish, occurring without changes in total leukocyte counts, are compatible with a limited modulation of basal hematological status rather than hematological disruption. Such responses may be relevant in intensive production systems, where fish are exposed to fluctuating environmental and microbial pressures. However, the biological meaning of these shifts and their relationship with disease outcomes require confirmation through adequately powered trials integrating functional immune markers, oxidative-stress endpoints, gut histology, microbiota profiling and pathogen-load measurements.

Finally, both *T. molitor* and *H. illucens* meals appear to be promising ingredients for juvenile Nile tilapia feeds. The choice between these insect meals should not be based solely on biological performance, but also on practical feasibility, including supply-chain availability, local regulations, environmental constraints, ingredient consistency, processing infrastructure and production costs. From this perspective, insect meals should be evaluated as regionalized solutions within aquafeed systems. Future studies should therefore couple biological performance with techno-economic and life-cycle assessments to support realistic implementation in commercial aquaculture.

## Figures and Tables

**Table 1:** Ingredient composition and calculated nutritional composition of the experimental diets for Nile tilapia juveniles.

Diet	CON	TM	HI	TMHI
Ingredient composition (g kg^−1^)				
Insect meal ^1^	0	100	100	100
Poultry by-product meal	109	38.6	40	42.5
Meat and bone meal	70	70	70	70
Blood meal	50	33.5	32.8	30
Soybean meal	310	320	303.7	314.1
Wheat bran	100	100	100	100
Broken rice	80	80	80	80
Corn	200	200	190	191
Corn starch	40	40	40	40
Soybean oil	26.5	0	28.6	17.3
Dicalcium phosphate	8.4	10	9.2	9.3
DL-methionine	1	2	1	0.7
Vitamin–mineral premix ^2^	5	5	5	5
Calculated composition				
Crude protein (%)	31.5	31.5	31.5	31.5
Digestible energy (kJ kg^−1^)	13,288	13,531	13,572	13,568
Ether extract (%)	6.7	6.7	6.7	6.7
Ash (%)	7.7	7.1	7.7	7.3
Crude fiber (%)	3.4	3.4	3.2	3.3
Nitrogen-free extract (%)	40.6	41	40.8	41.3
Calcium (%)	1.4	1.33	1.37	1.32
Available phosphorus (%)	0.81	0.83	0.84	0.82
Lysine (%)	1.72	1.76	1.79	1.79
Methionine (%)	0.54	0.65	0.54	0.56

^1^ Insect meal was included at 10% diet on a dry matter basis. CON: diet without insect ingredients; TM: diet containing 10% *Tenebrio molitor* larvae meal; HI: diet containing 10% *Hermetia illucens* larvae meal; TMHI: diet containing 5% *T. molitor* + 5% *H. illucens* larvae meals. ^2^ Vitamin–mineral premix composition (per kg of premix): choline 100 g; vitamin A 1,750,000 IU; vitamin D_3_ 375,000 IU; vitamin E 20,000 IU; vitamin K_3_ 500 mg; vitamin B_1_ 2000 mg; vitamin B_2_ 2500 mg; vitamin B_6_ 2500 mg; vitamin B_12_ 5000 µg; niacin 8750 mg; pantothenic acid 7500 mg; folic acid 625 mg; biotin 50 mg; vitamin C 37.5 g; inositol 12.5 g; iron 15 g; copper 1250 mg; manganese 3750 mg; zinc 17.5 g; cobalt 50 mg; iodine 100 mg; selenium 75 mg.

**Table 2:** Crude protein and total lipids (dry matter basis) of insect larvae meals.

Identification *	Crude Protein (%) (Mean ± SD **)	*p*	Total Lipids (%) (Mean ± SD **)	*p*
TMM	49.9 ± 0.9	NR	35.5 ± 0.2	NR
HIM	57.9 ± 0.2	NR	7.7 ± 0.2	NR

* TMM: *Tenebrio molitor* larvae meal; HIM: *Hermetia illucens* larvae meal. ** SD: standard deviation. NR: not reported (no statistical test performed).

**Table 3:** Total fatty acid classes (dry matter basis, g 100 g^−1^) of insect larvae meals.

Identification *	Saturated	Monounsaturated	ω-3	ω-6
TMM	7.97	16.63	0.26	8.49
HIM	4.87	1.38	0.02	0.71

* TMM: *Tenebrio molitor* larvae meal; HIM: *Hermetia illucens* larvae meal.

**Table 4:** Amino acid composition (dry matter basis, g 100 g^−1^ protein) of insect larvae meals.

Amino Acid	TMM *	HIM *
Aspartic acid	4.15	5.56
Glutamic acid	5.72	6.51
Serine	2.28	2.41
Glycine	3.13	3.86
Histidine	1.50	1.83
Arginine	2.81	3.11
Threonine	2.04	2.25
Alanine	3.59	3.50
Proline	2.85	3.03
Tyrosine	3.79	3.78
Valine	3.24	3.56
Methionine	0.80	1.12
Cystine	0.29	0.24
Isoleucine	2.20	2.40
Leucine	3.68	3.91
Phenylalanine	1.93	2.53
Lysine	2.71	3.51
Tryptophan	0.22	0.42

* TMM: *Tenebrio molitor* larvae meal; HIM: *Hermetia illucens* larvae meal.

**Table 5:** Growth performance of Nile tilapia juveniles fed diets containing insect larvae meals.

Diet	Initial BW (g)	Final BW (g)	WG (g)	SGR (% day^−1^)	Feed Intake (g)	FCR
CON	28.83 ± 0.64	248.31 ± 13.31	219.48 ± 13.23	2.76 ± 0.07	262.34 ± 15.74	1.20 ± 0.03
TM	28.28 ± 0.28	254.99 ± 10.77	226.71 ± 10.50	2.82 ± 0.04	260.58 ± 3.94	1.15 ± 0.05
HI	28.75 ± 0.75	252.97 ± 19.14	224.21 ± 19.05	2.79 ± 0.10	276.24 ± 13.38	1.25 ± 0.04
TMHI	29.22 ± 0.32	260.36 ± 6.35	231.14 ± 6.05	2.80 ± 0.02	270.63 ± 2.71	1.17 ± 0.02
*p*-value	0.2802	0.7388	0.7464	0.7110	0.3043	0.0525

CON: diet without insect ingredients; TM: diet containing 10% *Tenebrio molitor* larvae meal; HI: diet containing 10% *Hermetia illucens* larvae meal; TMHI: diet containing 5% *T. molitor* + 5% *H. illucens* larvae meals. BW: body weight; WG: weight gain; SGR: specific growth rate; FCR: feed conversion ratio. Values are means (*n* = 3 tanks per treatment; 20 fish per tank at stocking) ± standard deviation. The tank was considered the experimental unit.

**Table 6:** Survival and somatic indices of Nile tilapia juveniles fed diets containing *Tenebrio molitor* and *Hermetia illucens* larvae meals.

Diet	Survival (%)	Condition Factor	VSI (%)	HSI (%)	LSI (%)
CON	96.7 ± 5.8	2.05 ± 0.12	10.36 ± 0.24	2.38 ± 0.17	1.95 ± 0.34
TM	100.0 ± 0.0	2.19 ± 0.05	10.88 ± 0.51	1.99 ± 0.43	2.23 ± 0.32
HI	93.3 ± 5.8	2.09 ± 0.11	10.34 ± 2.01	2.14 ± 0.83	1.72 ± 0.49
TMHI	100.0 ± 0.0	2.10 ± 0.06	10.96 ± 0.74	1.81 ± 0.14	2.40 ± 0.06
*p*-value	0.7797	0.3832	0.8487	0.5478	0.0544

CON: diet without insect ingredients; TM: diet containing 10% *Tenebrio molitor* larvae meal; HI: diet containing 10% *Hermetia illucens* larvae meal; TMHI: diet containing 5% *T. molitor* + 5% *H. illucens* larvae meals. VSI: viscerosomatic index; HSI: hepatosomatic index; LSI: liposomatic index. Values are means (*n* = 3 tanks per treatment) ± standard deviation. Somatic indices were determined from three fish per tank, and tank means were used for statistical analysis. The tank was considered the experimental unit.

**Table 7:** Apparent digestibility coefficients (ADC, %) of crude protein and ether extract of the experimental diets and test ingredients (insect meals).

Diet	Diets—Test Diets		Ingredients—Insect Meals	
	ADC-CP (%)	ADC-EE (%)	ADC-CP (%)	ADC-EE (%)
CON	86.95 ± 0.71 ᵃ	87.22 ± 1.57	—	—
TM	83.60 ± 1.87 ᵇ	87.66 ± 1.42	75.79 ± 6.22 ᵇ	88.70 ± 4.73
HI	87.31 ± 0.87 ᵃ	86.76 ± 1.66	88.16 ± 2.90 ᵃ	85.69 ± 5.52
TMHI	85.71 ± 0.42 ᵃ	88.95 ± 0.91	82.83 ± 1.41 ᵃᵇ	93.48 ± 2.93
*p*-value	0.0134	0.9132	0.0265	0.8343

CON: diet without insect ingredients; TM: diet containing 10% *Tenebrio molitor* larvae meal; HI: diet containing 10% *Hermetia illucens* larvae meal; TMHI: diet containing 5% *T. molitor* + 5% *H. illucens* larvae meals. ADC-CP: apparent digestibility coefficient of crude protein; ADC-EE: apparent digestibility coefficient of ether extract. Values are means (*n* = 3 tanks per treatment; 30 fish per tank) ± standard deviation. Fecal samples from three collection cycles were pooled by experimental unit before chemical analysis. The tank was considered the experimental unit. Different superscript letters within the same column indicate significant differences (*p* < 0.05, Tukey’s test).

**Table 8:** Red blood cell (erythrocytes) and total leukocyte counts of Nile tilapia juveniles fed diets containing *Tenebrio molitor* and *Hermetia illucens* larvae meals.

Diet	Erythrocytes (×10^3^ µL^−1^)	Leukocytes (×10^3^ µL^−1^)
CON	2461.6 ± 216.5	26.58 ± 3.28
TM	2536.7 ± 270.3	25.71 ± 3.70
HI	2307.5 ± 284.8	26.37 ± 4.73
TMHI	2612.5 ± 369.3	31.18 ± 9.31
*p*-value	0.3332	0.3616

CON: diet without insect ingredients; TM: diet containing 10% *Tenebrio molitor* larvae meal; HI: diet containing 10% *Hermetia illucens* larvae meal; TMHI: diet containing 5% *T. molitor* + 5% *H. illucens* larvae meals. Values are means ± SD of six fish per diet (two fish per tank; three tanks per treatment). Fish were treated as subsamples within tanks, and tank nested within diet was included as a random effect in the statistical model.

**Table 9:** Differential leukocyte counts (%) of Nile tilapia juveniles fed diets containing *Tenebrio molitor* and *Hermetia illucens* larvae meals.

Diet	Neutrophils (%)	Lymphocytes (%)	Monocytes (%)	Eosinophils (%)	Basophils (%)
CON	32.33 ± 5.75 ᵃ	55.33 ± 3.62 ᵇ	12.00 ± 4.20	0.17 ± 0.41	0.17 ± 0.41
TM	26.33 ± 3.88 ᵃᵇ	61.83 ± 4.49 ᵃᵇ	11.67 ± 2.34	0.17 ± 0.41	0.00 ± 0.00
HI	22.50 ± 5.57 ᵇ	66.33 ± 6.41 ᵃ	10.33 ± 3.01	0.33 ± 0.52	0.50 ± 0.55
TMHI	21.83 ± 8.11 ᵇ	65.00 ± 7.77 ᵃ	13.00 ± 2.10	0.16 ± 0.41	0.00 ± 0.00
*p*-value	0.0247	0.0172	0.5101	0.8832	0.0628

CON: diet without insect ingredients; TM: diet containing 10% *Tenebrio molitor* larvae meal; HI: diet containing 10% *Hermetia illucens* larvae meal; TMHI: diet containing 5% *T. molitor* + 5% *H. illucens* larvae meals. Values are means ± SD of six fish per diet (two fish per tank; three tanks per treatment). Fish were treated as subsamples within tanks, and tank nested within diet was included as a random effect in the statistical model. Different superscript letters within the same column indicate significant differences (*p* < 0.05, Tukey’s test).

**Table 10:** Cumulative mortality and survival of Nile tilapia juveniles after intraperitoneal challenge with *Streptococcus agalactiae* (pooled data from Infection 1 and Infection 2).

Diet	*n* (Total)	Dead (*n*)	Survivors (*n*)	Cumulative Mortality (%) [95% CI]	Survival (%)	Observation Period (Days)
CON	46	30	16	65.2 [50.8–77.3]	34.8	13
TM	46	24	22	52.2 [38.1–65.9]	47.8	13
HI	46	23	23	50.0 [36.1–63.9]	50.0	13
TMHI	46	28	18	60.9 [46.5–73.6]	39.1	13

CON: diet without insect ingredients; TM: diet containing 10% *Tenebrio molitor* larvae meal; HI: diet containing 10% *Hermetia illucens* larvae meal; TMHI: diet containing 5% *T. molitor* + 5% *H. illucens* larvae meals. Values are pooled outcomes from two independent challenge blocks (Infection 1: *n* = 24 fish per diet; Infection 2: *n* = 22 fish per diet; total *n* = 46 per diet). Percentages are based on counts (Dead/n). The 95% confidence interval (CI) for cumulative mortality was calculated using the Wilson method. Kaplan–Meier curves were derived from daily deaths (Table S2); log-rank test among diets: χ^2^(3) = 1.907, *p* = 0.592.

## Data Availability

The data supporting the findings of this study are available from the corresponding author upon reasonable request.

## Supplementary Materials

The following supporting information can be downloaded at: https://www.mdpi.com/article/10.3390/insects17080762/s1, Figure S1. Experimental design and methodological workflow. Table S1. Cumulative mortality by diet in each independent challenge block and pooled dataset. Table S2. Daily deaths (n) and cumulative mortality (%) of Nile tilapia juveniles after intraperitoneal challenge with *Streptococcus agalactiae* (pooled Infection 1 + Infection 2; *n* = 46 per diet).

## Abbreviations

The following abbreviations are used in this manuscript:

ADC	Apparent Digestibility Coefficient
ADC-CP	Apparent Digestibility Coefficient of Crude Protein
ADC-EE	Apparent Digestibility Coefficient of Ether Extract
AOAC	Association of Official Analytical Chemists
BHI	Brain Heart Infusion
BW	Body Weight
CFU	Colony-Forming Units
CON	Diet without insect ingredients
Cr_2_O_3_	Chromium(III) Oxide
EDTA	Ethylenediaminetetraacetic Acid
FAME	Fatty Acid Methyl Esters
FCR	Feed Conversion Ratio
FI	Feed Ingestion
HI	Diet containing 10% *Hermetia illucens* larvae meal
HIM	*Hermetia illucens* larvae meal
HSI	Hepatosomatic Index
LD_50_	Median Lethal Dose
LSI	Liposomatic Index
PER	Protein Efficiency Ratio
SGR	Specific Growth Rate
TM	Diet containing 10% *Tenebrio molitor* larvae meal
TMHI	Diet containing 5% *T. molitor* + 5% *H. illucens* larvae meals
TMM	*Tenebrio molitor* larvae meal
