# Supplementary material for: Tenebrio molitor and Hermetia illucens Larvae Meals in Juvenile Nile Tilapia Diets: Performance, Digestibility and Hematological Responses Under Streptococcus agalactiae Challenge
Source: Insects. 2026 Jul 25;17(8):762. doi: 10.3390/insects17080762 (PMC13513066; doi:10.3390/insects17080762)
Supplement: Supplementary file 1 [file insects-17-00762-s001.zip › insects-4280339-supplementary.pdf]

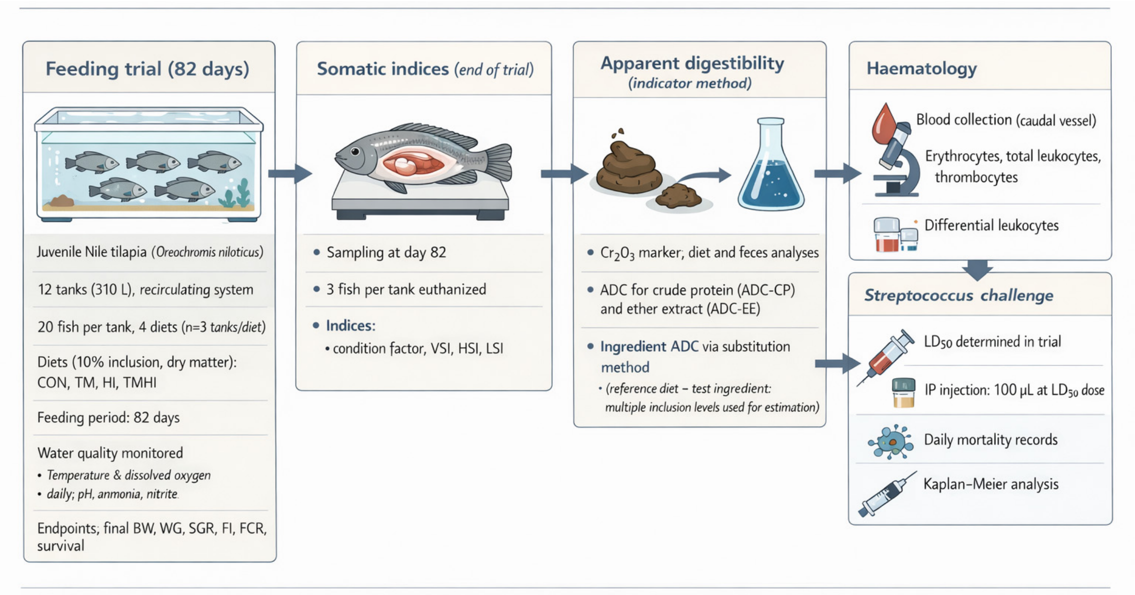

Figure S1. Experimental design and methodological workflow.

CON: diet without insect ingredients; TM: diet containing 100 g kg^{-1} *Tenebrio molitor* larvae meal; HI: diet containing 100 g kg^{-1} *Hermetia illucens* larvae meal; TMHI: diet containing 50 g kg^{-1} *T. molitor* + 50 g kg^{-1} *H. illucens* larvae meals; BW: body weight; WG: weight gain; SGR: specific growth rate; FI: feed ingestion; FCR: feed conversion ratio; VSI: viscerosomatic index; HSI: hepatosomatic index; LSI: liposomatic index; ADC: apparent digestibility coefficient; ADC-CP: apparent digestibility coefficient of crude protein; ADC-EE: apparent digestibility coefficient of ether extract; LD_{50}: median lethal dose.

Table S1. Cumulative mortality by diet in each independent challenge block and pooled dataset.

Group	Infection 1 (n=24) deaths	Infection 1 mortality (%)	Infection 2 (n=22) deaths	Infection 2 mortality (%)	Pooled (n=46) deaths	Pooled mortality (%)
CON	15	62.5	15	68.2	30	65.2
TM	12	50.0	12	54.5	24	52.2
HI	12	50.0	11	50.0	23	50.0
TMHI	15	62.5	13	59.1	28	60.9
CP (positive control)	15	62.5	15	68.2	30	65.2

CN (negative control)	4	16.7	0	0.0	4	8.7

Infection 1 and Infection 2 were conducted under the same protocol at different dates. Values are deaths/total fish per group and corresponding cumulative mortality (%).

Table S2. Daily deaths (n) and cumulative mortality (%) of Nile tilapia juveniles after intraperitoneal challenge with *Streptococcus agalactiae* (pooled Infection 1 + Infection 2; *n* = 46 per diet).

**Day post-challenge**	**CON (dead n)**	**CON (cum. mort. %)**	**TM (dead n)**	**TM (cum. mort. %)**	**HI (dead n)**	**HI (cum. mort. %)**	**TMHI (dead n)**	**TMHI (cum. mort. %)**
0	0	0.0	0	0.0	0	0.0	0	0.0
1	0	0.0	0	0.0	0	0.0	0	0.0
2	0	0.0	1	2.2	2	4.3	1	2.2
3	10	21.7	8	19.6	9	23.9	8	19.6
4	9	41.3	7	34.8	6	37.0	8	37.0
5	3	47.8	3	41.3	2	41.3	7	52.2
6	1	50.0	2	45.7	1	43.5	1	54.3
7	1	52.2	1	47.8	1	45.7	1	56.5
8	2	56.5	0	47.8	0	45.7	0	56.5
9	0	56.5	1	50.0	0	45.7	0	56.5
10	1	58.7	0	50.0	1	47.8	1	58.7
11	2	63.0	0	50.0	1	50.0	1	60.9
12	1	65.2	1	52.2	0	50.0	0	60.9

13	0	65.2	0	52.2	0	50.0	0	60.9

CON: diet without insect ingredients; TM: diet containing 100 g kg^{-1} *Tenebrio molitor* larvae meal; HI: diet containing 100 g kg^{-1} *Hermetia illucens* larvae meal; TMHI: diet containing 50 g kg^{-1} *T. molitor* + 50 g kg^{-1} *H. illucens* larvae meals. Daily values are based on pooled deaths from two independent challenge blocks (Infection 1 + Infection 2), total $n = 46$ fish per diet. "cum. mort. (%)" was calculated as (cumulative deaths / 46) × 100.
